# One-pot three-component synthesis of novel spirooxindoles with potential cytotoxic activity against triple-negative breast cancer MDA-MB-231 cells

**DOI:** 10.1080/14756366.2017.1417276

**Published:** 2017-12-28

**Authors:** Wagdy M. Eldehna, Dina H. EL-Naggar, Ahmed R. Hamed, Hany S. Ibrahim, Hazem A. Ghabbour, Hatem A. Abdel-Aziz

**Affiliations:** a Department of Pharmaceutical Chemistry, Faculty of Pharmacy, Kafrelsheikh University, Kafr El-Sheikh, Egypt;; b Department of Applied Organic Chemistry, National Research Center, Giza, Egypt;; c Department of Phytochemistry, National Research Center, Giza, Egypt;; d Biology Unit, Central Laboratory of the Pharmaceutical & Drug Industries Research Division, National Research Center, Giza, Egypt;; e Department of Pharmaceutical Chemistry, Faculty of Pharmacy, Egyptian Russian University, Badr City, Cairo, Egypt;; f Department of Medicinal Chemistry, Faculty of Pharmacy, Mansoura University, Mansoura, Egypt;; g Department of Pharmaceutical Chemistry, College of Pharmacy, King Saud University, Riyadh, Saudi Arabia

**Keywords:** Triple-negative breast cancer, spirooxindoles, anti-proliferative activity, apoptosis, EGFR

## Abstract

Triple-negative breast cancer (TNBC) is a highly aggressive malignancy with limited treatment options due to its heterogeneity and the lack of well-defined molecular targets. In our endeavour towards the development of novel anti-TNBC agents, herein we report a one-pot three-component synthesis of novel spirooxindoles **6a–p**, and evaluation of their potential anti-proliferative activity towards TNBC MDA-MB-231 cells. Spirooxindoles **6a**, **6e** and **6i** emerged as the most potent analogues with IC_50_ = 6.70, 6.40 and 6.70 µM, respectively. Compounds **6a** and **6e** induced apoptosis in MDA-MB-231 cells, as evidenced by the up-regulation of the Bax and down-regulation of the Bcl-2, besides boosting caspase-3 levels. Additionally, **6e** displayed significant increase in the percent of annexin V-FITC positive apoptotic cells from 1.34 to 44%. Furthermore, spirooxindoles **6e** and **6i** displayed good inhibitory activity against EGFR (IC_50_ = 120 and 150 nM, respectively). Collectively, these data demonstrated that **6e** might be a potential lead compound for the development of effective anti-TNBC agents.

## Introduction

Breast cancer is the fifth most common cause of death in women world-wide. In fact, it represents about 12% of all new cancer cases and 25% of all cancers in women with nearly 1.7 million new cases diagnosed in 2012^1^. Routine breast cancer case showed an expression of three distinctive receptors which are oestrogen receptor (ER), progesterone receptor (PR) and human epidermal growth factor 2 receptor (Her2). About 15–20% of the women diagnosed with breast cancer lacked in overexpression of these three receptors (ER, PR and Her2)^2^. This case is known as triple-negative breast cancer (TNBC) and it does not respond to normal protocol for the treatment of normal breast cancer. As a result, TNBC is responsible for high incident among the total percent of deaths regarded to breast cancer^3^^,^^4^. Unfortunately, TNBC patients were subjected to traditional cytotoxic therapies during their treatment protocol as till now there is no approved targeted cytotoxic drug for TNBC^5^.

**Figure 1.**
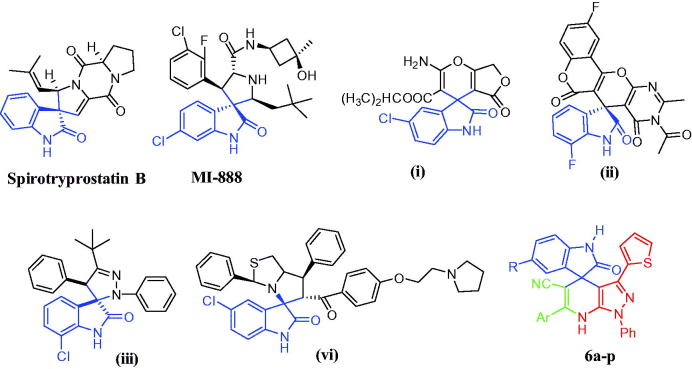
Structures of some reported anticancer spirooxindoles and target spirooxindoles **6a–p**.

On the other hand, spirooxindole is considered as a scaffold of interest to produce many derivatives with anticancer activity^6^. For example, Spirotryprostatins B (Figure 1), naturally isolated alkaloids from *Aspergillus fumigatus*, inhibit the cell cycle progression of tsFT210 cells at G2/M phase with IC_50_ = 14.0 µM^7^. Other synthetic spirooxindole derivative (MI-888) (Figure 1) in preclinical trials on xenograft models as mdm2-p53 inhibitor (*Ki* = 0.44 nM)^8^. Regarding cytotoxicity of some spirooxindole derivatives against TNBC cell lines, compound (**i**) (Figure 1) showed cytotoxic activity with IC_50_ = 11 µM against MDA-MB-231 cancer cell line^9^, while spirooxindole–pyranopyrimidine derivative (**ii**) (Figure 1) possessed cytotoxicity against MDA cell lines with IC_50_ = 6.9 µM^10^. Moreover, compound (**iii**) exhibited cytotoxic activity against ordinary breast cancer (MCF-7; IC_50_ = 8.6 µM) and TNBC (MDA-MB-231; IC_50_ = 6.4 µM)^11^ while compound (**iv**), Figure 1, displayed cytotoxicity against MDA-MB-231 with IC_50_ = 4.2 µM). Unfortunately, there is no trial till now to investigate the mechanism of action of spirooxindole derivative with cytotoxic activity against TNBC cell lines.

Based on the aforementioned findings and as a continuation of our research program on the development of novel effective anti-TNBC candidates^12–14^, it was thought worthwhile to extend our investigations to probe for novel spirooxindoles possessing promising anti-proliferative activity towards TNBC. In the present work we report the synthesis of a novel series of spirooxindoles **6a–p**, Figure 1, and their *in vitro* efficacy against the proliferation of the aggressive TNBC MDA-MB-231 cell line. In addition, spirooxindoles were further investigated regarding their potential apoptotic induction and their effects on cell cycle progression in the MDA-MB-231 cancer cells to acquire a perception for the mechanism of the antitumor activity of target spirooxindoles. Since the epidermal growth factor receptor (EGFR) is frequently overexpressed in TNBC^15^^,^^16^, the most potent spirooxindoles in this study will be assayed for their potential inhibitory activity towards EGFR.

## Materials and methods

### Chemistry

Melting points were measured with a Stuart melting point apparatus and were uncorrected. Infrared (IR) Spectra were recorded as KBr disks using Schimadzu FT-IR 8400 S spectrophotometer. Mass spectral data are given by GCMS-QP1000 EX spectrometer at 70 e.V. NMR Spectra were recorded on a Varian Mercury NMR spectrometer. ^1^H spectrum was run at 400 MHz and ^13 ^C spectrum was run at 100 MHz in deuterated dimethylsulfoxide (DMSO-d_6_). Chemical shifts are expressed in values (*ppm*) using the solvent peak as internal standard. All coupling constant (*J*) values are given in hertz. The abbreviations used are as follows: s, singlet; d, doublet; m, multiplet. Elemental analyses were carried out at the Regional Center for Microbiology and Biotechnology, Al-Azhar University, Cairo, Egypt. Analytical thin layer chromatography (TLC) on silica gel plates containing UV indicator was employed routinely to follow the course of reactions and to check the purity of products. Some representative NMR charts are provided in the Supplementary material.

#### Synthesis of 3-oxo-3-phenylpropanenitriles (3)

To ethyl benzoates **2a–d** (100 mmol) and acetonitrile (4.1 ml, 100 mol) in dry benzene (250 ml) and dimethylformamide (10 ml), sodium hydride (4.8 g, 60%) was added. The reaction mixture was heated under reflux for 4 h then allowed to cool to room temperature. The solid formed was collected by filtration, washed with ether and dried. This solid was dissolved in water and then neutralized with conc. HCl to pH 7. The precipitated product was collected by filtration, washed with water and dried. Recrystallization from ethanol gave compounds **3**^17^.

#### Synthesis of 1-phenyl-3-(thiophen-2-yl)-1H-pyrazol-5-amine (5)

To a stirred solution of 3-oxo-3-(thiophen-2-yl)propanenitrile **4** (1.5 g, 10 mmol) in ethyl alcohol (15 ml), phenylhydrazine (1.96 ml, 2.16 g, 20 mmol) was added. The reaction mixture was heated under reflux for 1 h. The solid product obtained upon cooling was filtered off, washed with cold water and recrystallized from methanol to furnish compound **5** (yield 65%), m.p. 113–115 °C; IR (KBr, *ν* cm^−1^): 3351 (NH_2_); ^1^H NMR (DMSO-d_6_) *δ* (*ppm*): 5.51 (s, 2H, NH_2_), 5.86 (s, 1H, H4-pyrazole), 7.19 (t, 1H, H4-thiophene, *J* = 4.8 Hz), 7.34–7.65 (m, 7H, Ar-H); ^13 ^C NMR (DMSO-d_6_) *δ ppm*: 87.54, 123.48 (2 C), 124.33, 125.23, 126.88, 127.99, 129.46 (2 C), 137.54, 139.46, 146.38, 148.69; Anal. calcd. For C_13_H_11_N_3_S: C, 64.71; H, 4.59; N, 17.41; Found C, 64.92; H, 4.54; N, 17.33.

#### General procedure for the synthesis of target compounds (6a–p)

A mixture of 1*H*-indole-2,3-diones **1a–d** (1 mmol) with an equimolar amount of 3-oxo-3-phenylpropanenitriles **3a–d** and 1-phenyl-3-(thiophen-2-yl)-1*H*-pyrazol-5-amine **5** in 15 ml of HOAc/H_2_O (1:1 v/v) at 120 °C was stirred for 8–11 h (the reaction progress was monitored by TLC). Upon completion, the reaction mixture was cooled to room temperature. The solid formed was filtered, dried and crystallized from acetonitrile to produce compounds **6a–p**.

#### 2-Oxo-1',6'-diphenyl-3'-(thiophen-2-yl)-1',7'-dihydrospiro[indoline-3,4'-pyrazolo[3,4-b]pyridine]-5'-carbonitrile (**6a**)

Yield 68%, m.p. 295–297 °C; IR (KBr, *ν* cm^−1^): 3227 (NH), 2205 (C≡N), 1709 (C=O); ^1^H NMR (DMSO-d_6_) *δ* (*ppm*): 6.21 (d, 1H, Ar-H, *J* = 2.8 Hz), 6.82 (dd, 1H, Ar-H, *J* = 4.8, 3.6 Hz), 6.92 (d, 1H, Ar-H, *J* = 8.0 Hz), 7.05 (t, 1H, Ar-H, *J* = 7.2 Hz), 7.29 (d, 1H, Ar-H, *J* = 7.6 Hz), 7.32 (d, 1H, Ar-H, *J* = 7.6 Hz), 7.40 (d, 1H, Ar-H, *J* = 5.2 Hz), 7.44–7.62 (m, 8H, Ar-H), 7.68 (d, 2H, Ar-H, *J* = 7.6 Hz), 10.45 (s, 1H, NH, D_2_O exchangeable), 10.74 (s, 1H, NH, D_2_O exchangeable); ^13 ^C NMR (DMSO-d_6_) *δ ppm*: 51.83, 83.74, 97.41, 110.47, 118.66, 123.28, 124.69 (2 C), 125.44, 125.80, 126.98, 127.62, 128.44, 128.89, 129.35 (2 C), 129.60, 129.90, 130.01 (2 C), 130.99, 133.78, 134.48, 135.64, 138.00, 139.66, 141.70, 142.97, 151.53, 178.02 (C=O); MS *m/z* [%]: 497 [M^+^, 26.91], 77 [100]; Anal. calcd. For C_30_H_19_N_5_OS: C, 72.42; H, 3.85; N, 14.08; Found C, 72.59; H, 3.79; N, 13.95.

#### 2-Oxo-1'-phenyl-3'-(thiophen-2-yl)-6'-(p-tolyl)-1',7'-dihydrospiro[indoline-3,4'-pyrazolo[3,4-b]pyridine]-5'-carbonitrile (6 b)

Yield 75%, m.p. 285–287 °C; IR (KBr, *ν* cm^−1^): 3317 (NH), 2206 (C≡N), 1712 (C=O); ^1^H NMR (DMSO-d_6_) *δ* (*ppm*): 2.33 (s, 3H, CH_3_), 6.15 (d, 1H, Ar-H, *J* = 3.2 Hz), 6.78 (dd, 1H, Ar-H, *J* = 4.8, 3.6 Hz), 6.87 (d, 1H, Ar-H, *J* = 8.0 Hz), 7.00 (t, 1H, Ar-H, *J* = 7.6 Hz), 7.27 (d, 2H, Ar-H, *J* = 8.4 Hz), 7.37–7.47 (m, 6H, Ar-H), 7.53 (t, 2H, Ar-H, *J* = 8.0 Hz), 7.64 (d, 2H, Ar-H, *J* = 8.0 Hz), 10.33 (s, 1H, NH, D_2_O exchangeable), 10.68 (s, 1H, NH, D_2_O exchangeable); ^13 ^C NMR (DMSO-d_6_) *δ ppm*: 21.37 (CH_3_), 51.76, 82.85, 97.40, 110.40, 132.22, 124.61 (2 C), 125.35, 125.75, 126.93, 127.59, 128.36, 129.24 (2 C), 129.33 (2 C), 129.54, 129.85 (2 C), 129.94, 130.88, 134.48, 135.63, 138.00, 139.64, 140.84, 141.68, 142.90, 151.46, 177.99 (C=O); MS *m/z* [%]: 511 [M^+^, 16.08], 482 [100]; Anal. calcd. For C_31_H_21_N_5_OS: C, 72.78; H, 4.14; N, 13.69; Found C, 72.54; H, 4.11; N, 13.81.

#### 6'-(4-Methoxyphenyl)-2-oxo-1'-phenyl-3'-(thiophen-2-yl)-1',7'-dihydrospiro[indoline-3,4'-pyrazolo[3,4-b]pyridine]-5'-carbonitrile (6c)

Yield 75%, m.p. 296–298 °C; IR (KBr, *ν* cm^−1^): 3361 (NH), 2206 (C≡N), 1715 (C=O); ^1^H NMR (DMSO-d_6_) *δ* (*ppm*): 3.81 (s, 3H, OCH_3_), 6.19 (d, 1H, Ar-H, *J* = 3.6 Hz), 6.81 (dd, 1H, Ar-H, *J* = 4.8, 3.6 Hz), 6.91 (d, 1H, Ar-H, *J* = 7.6 Hz), 7.03–7.06 (m, 3H, Ar-H), 7.28–7.32 (m, 2H, Ar-H), 7.40 (d, 1H, Ar-H, *J* = 4.8 Hz), 7.46 (t, 1H, Ar-H, *J* = 7.6 Hz), 7.55–7.60 (m, 4H, Ar-H), 7.68 (d, 2H, Ar-H, *J* = 7.6 Hz), 10.29 (s, 1H, NH, D_2_O exchangeable), 10.70 (s, 1H, NH, D_2_O exchangeable); MS *m/z* [%]: 527 [M^+^, 18.5], 498 [100]; Anal. calcd. For C_31_H_21_N_5_O_2_S: C, 70.57; H, 4.01; N, 13.27; Found C, 70.78; H, 3.96; N, 13.38.

#### 6'-(4-Chlorophenyl)-2-oxo-1'-phenyl-3'-(thiophen-2-yl)-1',7'-dihydrospiro[indoline-3,4'-pyrazolo[3,4-b]pyridine]-5'-carbonitrile (6d)

Yield 70%, m.p. 279–281 °C; IR (KBr, *ν* cm^−1^): 3283 (NH), 2202 (C≡N), 1716 (C=O); ^1^H NMR (DMSO-d_6_) *δ* (*ppm*): 6.20 (dd, 1H, Ar-H, *J* = 3.6, 0.8 Hz), 6.81 (dd, 1H, Ar-H, *J* = 4.8, 3.6 Hz), 6.91 (d, 1H, Ar-H, *J* = 7.6 Hz), 7.04 (t, 1H, Ar-H, *J* = 7.6 Hz), 7.29–7.34 (m, 2H, Ar-H), 7.41 (dd, 1H, Ar-H, *J* = 4.8, 0.8 Hz), 7.45 (t, 1H, Ar-H, *J* = 7.6 Hz), 7.56–7.64 (m, 6H, Ar-H), 7.68 (d, 2H, Ar-H, *J* = 7.6 Hz), 10.44 (s, 1H, NH, D_2_O exchangeable), 10.75 (s, 1H, NH, D_2_O exchangeable); Anal. calcd. For C_30_H_18_ClN_5_OS: C, 67.73; H, 3.41; N, 13.16; Found C, 67.61; H, 3.46; N, 13.25.

#### 5-Chloro-2-oxo-1',6'-diphenyl-3'-(thiophen-2-yl)-1',7'-dihydrospiro[indoline-3,4'-pyrazolo[3,4-b]pyridine]-5'-carbonitrile (6e)

Yield 78%, m.p. 289–291 °C; IR (KBr, *ν* cm^−1^): 3205 (NH), 2206 (C≡N), 1708 (C=O); ^1^H NMR (DMSO-d_6_) *δ* (*ppm*): 6.33 (dd, 1H, Ar-H, *J* = 3.6, 0.8 Hz), 6.86 (dd, 1H, Ar-H, *J* = 5.2, 3.6 Hz), 6.91 (d, 1H, Ar-H, *J* = 8.4 Hz), 7.33 (dd, 1H, Ar-H, *J* = 8.4, 2.4 Hz), 7.44–7.62 (m, 10H, Ar-H), 7.68 (d, 2H, Ar-H, *J* = 7.6 Hz), 10.52 (s, 1H, NH, D_2_O exchangeable), 10.87 (s, 1H, NH, D_2_O exchangeable); MS *m/z* [%]: 532 [M^+^, 5.28], 534 [M^+^+2, 1.81], 502 [100]; Anal. calcd. For C_30_H_18_ClN_5_OS: C, 67.73; H, 3.41; N, 13.16; Found C, 67.49; H, 3.44; N, 13.22.

#### 5-Chloro-2-oxo-1'-phenyl-3'-(thiophen-2-yl)-6'-(p-tolyl)-1',7'-dihydrospiro[indoline-3,4'-pyrazolo[3,4-b]pyridine]-5'-carbonitrile (6f)

Yield 75%, m.p. 286–288 °C; IR (KBr, *ν* cm^−1^): 3259 (NH), 2205 (C≡N), 1712 (C=O); ^1^H NMR (DMSO-d_6_) *δ* (*ppm*): 2.36 (s, 3H, CH_3_), 6.32 (dd, 1H, Ar-H, *J* = 4.0, 0.8 Hz), 6.86 (dd, 1H, Ar-H, *J* = 5.2, 4.0 Hz), 6.90 (d, 1H, Ar-H, *J* = 8.0 Hz), 7.30–7.35 (m, 3H, Ar-H), 7.42–7.47 (m, 3H, Ar-H), 7.48 (d, 2H, Ar-H, *J* = 8.0 Hz), 7.56 (t, 2H, Ar-H, *J* = 8.0 Hz), 7.67 (d, 2H, Ar-H, *J* = 8.0 Hz), 10.43 (s, 1H, NH, D_2_O exchangeable), 10.86 (s, 1H, NH, D_2_O exchangeable); Anal. calcd. For C_31_H_20_ClN_5_OS: C, 68.19; H, 3.69; N, 12.83; Found C, 67.92; H, 3.70; N, 12.89.

#### 5-Chloro-6'-(4-methoxyphenyl)-2-oxo-1'-phenyl-3'-(thiophen-2-yl)-1',7'-dihydrospiro[indoline-3,4'-pyrazolo[3,4-b]pyridine]-5'-carbonitrile (6 g)

Yield 68%, m.p. > 300 °C; IR (KBr, *ν* cm^−1^): 3213 (NH), 2206 (C≡N), 1708 (C=O); ^1^H NMR (DMSO-d_6_) *δ* (*ppm*): 3.78 (s, 3H, OCH_3_), 6.31 (d, 1H, Ar-H, *J* = 3.6 Hz), 6.83 (dd, 1H, Ar-H, *J* = 5.2, 3.6 Hz), 6.87 (d, 1H, Ar-H, *J* = 8.4 Hz), 7.02 (d, 2H, Ar-H, *J* = 8.4 Hz), 7.29 (dd, 1H, Ar-H, *J* = 8.0, 2.0 Hz), 7.34–7.63 (m, 7H, Ar-H), 7.66 (d, 2H, Ar-H, *J* = 8.0 Hz), 10.31 (s, 1H, NH, D_2_O exchangeable), 10.79 (s, 1H, NH, D_2_O exchangeable); ^13 ^C NMR (DMSO-d_6_) *δ ppm*: 55.84, 55.91 (OCH_3_), 82.13, 97.08, 111.89, 114.15, 114.56, 121.93, 124.71, 125.40, 125.64, 125.87, 127.12, 127.15, 127.63, 128.42, 129.82, 129.98, 130.14, 130.94, 131.47, 134.31, 137.35, 138.01, 138.49, 139.77, 140.64, 142.73, 161.45, 161.55, 177.90 (C=O); Anal. calcd. For C_31_H_20_ClN_5_O_2_S: C, 66.25; H, 3.59; N, 12.46; Found C, 66.41; H, 3.63; N, 12.35.

#### 5-Chloro-6'-(4-chlorophenyl)-2-oxo-1'-phenyl-3'-(thiophen-2-yl)-1',7'-dihydrospiro[indoline-3,4'-pyrazolo[3,4-b]pyridine]-5'-carbonitrile (6 h)

Yield 70%, m.p. 281–283 °C; IR (KBr, *ν* cm^−1^): 3324 (NH), 2202 (C≡N), 1715 (C=O); ^1^H NMR (DMSO-d_6_) *δ* (*ppm*): 6.31 (d, 1H, Ar-H, *J* = 3.2 Hz), 6.85 (t, 1H, Ar-H, *J* = 8.4 Hz), 6.90 (d, 1H, Ar-H, *J* = 8.4 Hz), 7.32 (dd, 1H, Ar-H, *J* = 8.4, 2.0 Hz), 7.38–7.57 (m, 7H, Ar-H), 7.62 (d, 2H, Ar-H, *J* = 8.4 Hz), 7.72–7.83 (m, 2H, Ar-H), 9.09 (s, 1H, NH, D_2_O exchangeable), 10.83 (s, 1H, NH, D_2_O exchangeable); ^13 ^C NMR (DMSO-d_6_) *δ ppm*: 55.32, 77.19, 96.50, 111.86, 112.87, 117.51, 122.10, 125.32, 125.99, 127.04, 127.14, 128.63, 128.80, 129.14, 129.77, 130.34, 131.30, 131.66, 132.09, 135.94, 136.52, 136.72, 140.51, 141.34, 142.70, 148.51, 150.43, 154.33, 159.62, 178.10 (C=O); Anal. calcd. For C_30_H_17_Cl_2_N_5_OS: C, 63.61; H, 3.03; N, 12.36; Found C, 63.38; H, 3.05; N, 12.49.

#### 5-Bromo-2-oxo-1',6'-diphenyl-3'-(thiophen-2-yl)-1',7'-dihydrospiro[indoline-3,4'-pyrazolo[3,4-b]pyridine]-5'-carbonitrile (6i)

Yield 75%, m.p. 275–277 °C; IR (KBr, *ν* cm^−1^): 3293 (NH), 2206 (C≡N), 1713 (C=O); ^1^H NMR (DMSO-d_6_) *δ* (*ppm*): 6.33 (d, 1H, Ar-H, *J* = 3.2 Hz), 6.87–6.91 (m, 2H, Ar-H), 7.44–7.62 (m, 11H, Ar-H), 7.69 (d, 2H, Ar-H, *J* = 7.6 Hz), 10.52 (s, 1H, NH, D_2_O exchangeable), 10.89 (s, 1H, NH, D_2_O exchangeable); ^13 ^C NMR (DMSO-d_6_) *δ ppm*: 52.02, 82.85, 96.88, 112.46, 114.88, 118.57, 124.69, 124.90, 125.47, 126.98, 127.22, 127.68, 128.54, 128.65, 128.86, 129.39, 129.85, 131.01, 132.83, 133.63, 134.29, 137.70, 137.95, 139.72, 140.53, 141.04, 141.89, 142.79, 151.92, 177.73 (C=O); MS *m/z* [%]: 576 [M^+^, 3.93], 77 [100]; Anal. calcd. For C_30_H_18_BrN_5_OS: C, 62.51; H, 3.15; N, 12.15; Found C, 62.63; H, 3.20; N, 12.03.

#### 5-Bromo-2-oxo-1'-phenyl-3'-(thiophen-2-yl)-6'-(p-tolyl)-1',7'-dihydrospiro[indoline-3,4'-pyrazolo[3,4-b]pyridine]-5'-carbonitrile (6j)

Yield 80%, m.p. 282–284 °C; IR (KBr, *ν* cm^−1^): 3207 (NH), 2204 (C≡N), 1708 (C=O); ^1^H NMR (DMSO-d_6_) *δ* (*ppm*): 2.33 (s, 3H, CH_3_), 6.30 (d, 1H, Ar-H, *J* = 2.8 Hz), 6.83–6.87 (m, 2H, Ar-H), 7.29 (d, 2H, Ar-H, *J* = 8.0 Hz), 7.41–7.50 (m, 6H, Ar-H), 7.55 (t, 2H, Ar-H, *J* = 8.0 Hz), 7.65 (d, 2H, Ar-H, *J* = 7.6 Hz), 10.39 (s, 1H, NH, D_2_O exchangeable), 10.82 (s, 1H, NH, D_2_O exchangeable); ^13 ^C NMR (DMSO-d_6_) *δ ppm*: 21.38 (CH_3_), 51.95, 82.44, 96.85, 112.41, 114.82, 124.82 (2 C), 125.38, 127.18, 127.66, 128.47, 128.59, 129.27, 129.31, 129.57, 129.59, 129.77, 129.82 (2 C), 130.73, 132.77, 134.30, 137.70, 137.94, 139.72, 140.87, 141.01, 142.73, 151.86, 177.72 (C=O); Anal. calcd. For C_31_H_20_BrN_5_OS: C, 63.06; H, 3.41; N, 11.86; Found C, 62.83; H, 3.48; N, 11.79.

#### 5-Bromo-6'-(4-methoxyphenyl)-2-oxo-1'-phenyl-3'-(thiophen-2-yl)-1',7'-dihydrospiro[indoline-3,4'-pyrazolo[3,4-b]pyridine]-5'-carbonitrile (6k)

Yield 81%, m.p. > 300 °C; IR (KBr, *ν* cm^−1^): 3387 (NH), 2202 (C≡N), 1716 (C=O); ^1^H NMR (DMSO-d_6_) *δ* (*ppm*): 3.78 (s, 3H, OCH_3_), 6.31 (d, 1H, Ar-H, *J* = 3.2 Hz), 6.83 (d, 2H, Ar-H, *J* = 8.4 Hz), 7.02 (d, 2H, Ar-H, *J* = 8.8 Hz), 7.40–7.55 (m, 8H, Ar-H), 7.66 (d, 2H, Ar-H, *J* = 8.0 Hz), 10.30 (s, 1H, NH, D_2_O exchangeable), 10.80 (s, 1H, NH, D_2_O exchangeable); ^13 ^C NMR (DMSO-d_6_) *δ ppm*: 51.96, 55.84 (OCH_3_), 82.08, 96.96, 112.41, 114.15 (2 C), 114.80, 118.83, 124.74 (2 C), 125.39, 125.62, 127.17, 127.65, 128.43, 128.56, 129.83 (2 C), 130.95 (2 C), 132.74, 134.31, 137.72, 137.99, 139.77, 141.04, 142.73, 151.57, 161.45, 177.76 (C=O); MS *m/z* [%]: 606 [M^+^, 2.24], 608 [M^+^+2, 1.20], 468 [100]; Anal. calcd. For C_31_H_20_BrN_5_O_2_S: C, 61.39; H, 3.32; N, 11.55; Found C, 61.57; H, 3.36; N, 11.39.

#### 5-Bromo-6'-(4-chlorophenyl)-2-oxo-1'-phenyl-3'-(thiophen-2-yl)-1',7'-dihydrospiro[indoline-3,4'-pyrazolo[3,4-b]pyridine]-5'-carbonitrile (6 l)

Yield 76%, m.p. 293–294 °C; IR (KBr, *ν* cm^−1^): 3225 (NH), 2206 (C≡N), 1715 (C=O); ^1^H NMR (DMSO-d_6_) *δ* (*ppm*): 6.30 (d, 1H, Ar-H, *J* = 3.3 Hz), 6.84 (d, 2H, Ar-H, *J* = 7.2 Hz), 7.43–7.54 (m, 3H, Ar-H), 7.57–7.63 (m, 7H, Ar-H), 7.66 (d, 2H, Ar-H, *J* = 8.1 Hz), 10.49 (s, 1H, NH, D_2_O exchangeable), 10.86 (s, 1H, NH, D_2_O exchangeable); Anal. calcd. For C_30_H_17_BrClN_5_OS: C, 58.98; H, 2.80; N, 11.46; Found C, 59.15; H, 2.77; N, 11.61.

#### 5-Methoxy-2-oxo-1',6'-diphenyl-3'-(thiophen-2-yl)-1',7'-dihydrospiro[indoline-3,4'-pyrazolo[3,4-b]pyridine]-5'-carbonitrile (6 m)

Yield 75%, m.p. 276–278 °C; IR (KBr, *ν* cm^−1^): 3319 (NH), 2202 (C≡N), 1716 (C=O); ^1^H NMR (DMSO-d_6_) *δ* (ppm): 3.68 (s, 3H, OCH_3_), 6.24 (d, 1H, Ar-H, *J* = 2.8 Hz), 6.79–6.90 (m, 3H, Ar-H), 7.38–7.62 (m, 10H, Ar-H), 7.65 (d, 2H, Ar-H, *J* = 7.6 Hz), 10.35 (s, 1H, NH, D_2_O exchangeable), 10.51 (s, 1H, NH, D_2_O exchangeable); ^13 ^C NMR (DMSO-d_6_) *δ ppm*: 56.00, 56.48 (OCH_3_), 83.92, 97.31, 110.96, 112.44, 114.72, 118.52, 122.10, 122.31, 124.68, 125.40, 126.90, 127.63, 128.39, 128.82, 129.06, 129.35, 129.84, 129.90, 129.97, 130.92, 133.86, 134.52, 134.95, 136.74, 137.97, 139.69, 142.94, 156.16, 177.83 (C=O); MS *m/z* [%]: 527 [M^+^, 16.84], 498 [93.20]; Anal. calcd. For C_31_H_21_N_5_O_2_S: C, 70.57; H, 4.01; N, 13.27; Found C, 70.41; H, 3.98; N, 13.35.

#### 5-Methoxy-2-oxo-1'-phenyl-3'-(thiophen-2-yl)-6'-(p-tolyl)-1',7'-dihydrospiro[indoline-3,4'-pyrazolo[3,4-b]pyridine]-5'-carbonitrile (6n)

Yield 78%, m.p. 268–269 °C; IR (KBr, *ν* cm^−1^): 3314 (NH), 2206 (C≡N), 1712 (C=O); ^1^H NMR (DMSO-d_6_) *δ* (ppm): 2.34 (s, 3H, CH_3_), 3.68 (s, 3H, OCH_3_), 6.26 (d, 1H, Ar-H, *J* = 2.8 Hz), 6.79–6.87 (m, 4H, Ar-H), 7.27 (d, 2H, Ar-H, *J* = 8.0 Hz), 7.37–7.48 (m, 4H, Ar-H), 7.52 (t, 2H, Ar-H, *J* = 8.0 Hz), 7.65 (d, 2H, Ar-H, *J* = 7.2 Hz), 10.21 (s, 1H, NH, D_2_O exchangeable), 10.45 (s, 1H, NH, D_2_O exchangeable); ^13 ^C NMR (DMSO-d_6_) *δ ppm*: 21.73 (CH_3_), 52.07, 56.00 (OCH_3_), 83.47, 97.31, 110.93, 112.43, 114.69, 118.77, 124.64 (2 C), 125.39, 126.89, 127.63, 128.34, 129.27, 129.31, 129.57, 129.77, 129.83 (2 C), 130.92, 134.61, 134.99, 136.78, 138.01, 139.68, 140.79, 142.94, 151.43, 156.15, 177.83 (C=O); Anal. calcd. For C_32_H_23_N_5_O_2_S: C, 70.96; H, 4.28; N, 12.93; Found C, 71.27; H, 4.22; N, 12.79.

#### 5-Methoxy-6'-(4-methoxyphenyl)-2-oxo-1'-phenyl-3'-(thiophen-2-yl)-1',7'-dihydrospiro[indoline-3,4'-pyrazolo[3,4-b]pyridine]-5'-carbonitrile (6o)

Yield 75%, m.p. 279–281 °C; IR (KBr, *ν* cm^−1^): 3361 (NH), 2204 (C≡N), 1710 (C=O); ^1^H NMR (DMSO-d_6_) *δ* (ppm): 3.70 (s, 3H, OCH_3_), 3.81 (s, 3H, OCH_3_), 6.27 (d, 1H, Ar-H, *J* = 2.8 Hz), 6.78–6.91 (m, 4H, Ar-H), 7.05 (d, 2H, Ar-H, *J* = 8.8 Hz), 7.41–7.48 (m, 2H, Ar-H), 7.53–7.60 (m, 4H, Ar-H), 7.70 (d, 2H, Ar-H, *J* = 7.6 Hz), 10.28 (s, 1H, NH, D_2_O exchangeable), 10.57 (s, 1H, NH, D_2_O exchangeable); ^13 ^C NMR (DMSO-d_6_) *δ ppm*: 52.32, 55.86 (OCH_3_), 56.02 (OCH_3_), 83.12, 97.47, 110.99, 112.44, 114.19, 114.72, 118.99, 122.03, 124.57, 125.45, 125.86, 126.90, 127.66, 128.34, 129.75, 129.88, 129.96, 130.97, 131.50, 134.60, 135.05, 136.84, 138.09, 139.78, 142.98, 151.21, 156.19, 161.44, 177.95 (C=O); Anal. calcd. For C_32_H_23_N_5_O_3_S: C, 68.93; H, 4.16; N, 12.56; Found C, 69.06; H, 4.19; N, 12.65.

#### 6'-(4-Chlorophenyl)-5-methoxy-2-oxo-1'-phenyl-3'-(thiophen-2-yl)-1',7'-dihydrospiro[indoline-3,4'-pyrazolo[3,4-b]pyridine]-5'-carbonitrile (6p)

Yield 80%, m.p. 255–257 °C; IR (KBr, *ν* cm^−1^): 3273 (NH), 2206 (C≡N), 1712 (C=O); ^1^H NMR (DMSO-d_6_) *δ* (ppm): 3.68 (s, 3H, OCH_3_), 6.31 (d, 1H, Ar-H, *J* = 2.8 Hz), 6.74–6.89 (m, 3H, Ar-H), 7.05 (d, 1H, Ar-H, *J* = 8.8 Hz), 7.05 (d, 1H, Ar-H, *J* = 8.8 Hz), 7.42 (d, 1H, Ar-H, *J* = 7.6 Hz), 7.50–7.67 (m, 2H, Ar-H), 7.69 (d, 2H, Ar-H, *J* = 8.0 Hz), 7.95 (d, 2H, Ar-H, *J* = 8.4 Hz), 8.23 (d, 2H, Ar-H, *J* = 8.4 Hz), 10.33 (s, 1H, NH, D_2_O exchangeable), 10.59 (s, 1H, NH, D_2_O exchangeable); Anal. calcd. For C_31_H_20_ClN_5_O_2_S: C, 66.25; H, 3.59; N, 12.46; Found C, 65.98; H, 3.61; N, 12.58.

### Biological evaluation

#### *In vitro* anti-proliferative activity assay

Synthetic spirooxindoles **6a–p** were tested for their anti-proliferative potency on TNBC cells (MDA-MB-231). Cells lines were maintained as monolayers in Dulbecco’s Modified Eagle’s Medium (DMEM) supplemented with 10% FBS, 2 mM l-glutamine, 100 U/ml penicillin and 100 µg/ml streptomycin sulfate. Cells were sub-cultured with trypsine/EDTA solution, counted with haemocytometer and plated onto 96-well plates (5000 cells/well) and left overnight to form a semi-confluent monolayer. We employed a modified method utilizing MTT (3-[4,5-dimethylthiazol-2-yl]-2,5-diphenyltetrazolium bromide dye (Carbosynth, UK) that is based on the reduction of the dye by mitochondrial dehydrogenases of metabolically active cells to insoluble formazan crystals^18^^,^^19^. Briefly, cell monolayers were treated in quadrates with vehicle (DMSO, 0.1% v/v), test samples or Adriamycin as positive control for an exposure time of 48 h. At the end of exposure, MTT solution in PBS (5 mg/ml) was then added to all wells including no cell blank and left to incubate for 90 min^20^. The formation of formazan crystals were visually confirmed using phase contract microscopy. DMSO (100 µl/well) was added to dissolve the formazan crystals with shaking for 10 min after which the absorbance was read at 590 nm against no cell blanks on a FLuo Star Optima microplate reader (BMG Technologies, Germany). Cell proliferation was calculated comparing the OD values of the DMSO control wells and those of the samples represented as % proliferation to the control. Dose-response experiment was performed on samples producing ≥50% loss of cell proliferation using five serial 2-fold dilutions (50, 25, 12.5, 6.25 and 3.125 µM) of the sample. IC_50_ values (concentration of sample causing 50% loss of cell proliferation of the vehicle control) were calculated using non-linear regression curve fitting of the dose response plots on GraphPad Prism V.6.0 software (Graphpad Inc, San Diego, CA). Assessment of morphological changes of MDA-MB-231 cells following treatment with the most active hits were performed using phase contrast inverted microscope (Carl Zeiss Microscopy GmbH, Gottingen, Germany) and photomicrographs were taken using digital camera.

#### *In vitro* cytotoxic activity WI-38 cells (human lung fibroblast normal cell line)

WI-38 cells (normal breast cells), were obtained from American Type Culture Collection. The cells were propagated in DMEM supplemented with 10% heat-inactivated FBS (Hyclone), 10 μg/ml of insulin (Sigma), and 1% penicillin-streptomycin. All of the other chemicals and reagents were from Sigma, or Invitrogen. Cytotoxicity was determined using MTT assay following a reported procedure^18^. The 50% inhibitory concentration (IC_50_) was estimated from graphic plots of the dose response curve for each conc. using Graphpad Prism software (San Diego, CA).

#### Assay of the apoptosis markers (Bax, caspase-3 and Bcl-2) levels

The levels of the apoptotic markers (Bax, caspase-3) as well as the anti-apoptotic marker (Bcl-2) were assessed using ELISA colorimetric kits per the manufacturer’s protocol and referring to reported instructions^21^^,^^22^.

#### Cell cycle analysis

The MDA-MB-231 cells were treated with 6.40 μM of compound **6e** for 24 h. Then cells were washed twice with ice-cold phosphate buffered saline (PBS). Subsequently, the treated cells were collected by centrifugation, fixed in ice-cold 70% (v/v) ethanol, washed with PBS, re-suspended with 0.1 mg/mL RNase, stained with 40 mg/mL PI, and analyzed by flow cytometry using FACS Calibur (Becton Dickinson, BD, USA). The cell cycle distributions were calculated using CellQuest software (Becton Dickinson).

#### Annexin V–FITC apoptosis assay

Phosphatidylserine externalization was measured using Annexin V-FITC/PI apoptosis detection kit (BD Biosciences, San Jose, CA) according to the manufacturer's instructions, as reported earlier^22^. MDA-MB-231cells were treated with **6e** at defined concentrations for 24 h.

#### EGFR kinase ELISA assay

EGFR enzyme inhibition was measured using a BPS Biosciences Colorimetric 96-well EGFR assay kit (catalog no. 40321), according to the manufacturer's instructions. Percent inhibition was calculated by the comparison of compounds treated to control incubations. The concentration of the test compound causing 50% inhibition (IC_50_) was calculated from the concentration–inhibition response curve (triplicate determinations) and the data were compared with Erlotinib as standard EGFR inhibitor.

## Results and discussion

### Chemistry

**Scheme 1.**
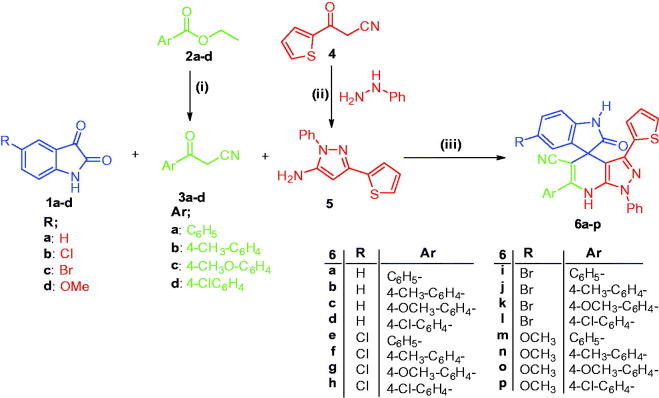
Synthesis of target compounds **6a–p**; *Reagents and conditions*: (**i**) CH_3_CN, DMF, NaH, benzene, reflux 4 h; (**ii**) Ethanol, phenylhydrazine, reflux 1 h; (**iii**) HOAc/H_2_O (1:1 v/v), heating at 120 °C, 8–11 h.

The proposed synthetic routes to prepare the target compounds are shown in Scheme 1. Synthesis was initiated in Scheme 1 by reacting ethyl benzoates **2a–d** and acetonitrile in dry benzene and DMF, in the presence of sodium hydride under reflux temperature to afford 3-oxo-3-phenylpropanenitriles **3a–d**. Also, synthesis of 1-phenyl-3-(thiophen-2-yl)-1*H*-pyrazol-5-amine **5** was accomplished *via* heterocyclocondensation of 3-oxo-3-(thiophen-2-yl)propanenitrile **4** with phenylhydrazine in refluxing absolute ethyl alcohol. Preparation of the target spirooxindoles **6a–p** was achieved *via* a one-pot three-component reaction of 1*H*-indole-2,3-diones **1a–d** with an equimolar amount of 3-oxo-3-phenylpropanenitriles **3a–d** and 1-phenyl-3-(thiophen-2-yl)-1*H*-pyrazol-5-amine **5** in hot HOAc/H_2_O (1:1 v/v), with 68–81% yield (Scheme 1).

Postulated structures of the newly synthesized spirooxindoles **6a–p** were in full agreement with their spectral and elemental analyses data. IR spectra of the latter products displayed absorption bands of the (NH) groups in the region 3205–3387 cm^−1^, in addition to the carbonyl bands in the region 1709–1716 cm^−1^ and C≡N bands in the region 2202–2206 cm^−1^. ^1^H NMR spectra of **6a–p** showed two singlet D_2_O-exchangeable signals attributable to two (NH) protons at range *δ* 10.09–10.52 and 10.45–10.89 *ppm*. In addition, the methoxy (-OCH_3_) protons of compounds **6c**, **6 g**, **6k** and **6 m–p** were displayed as singlet signals in the range *δ* 3.68–3.81 *ppm*, whereas, the methyl (-CH_3_) protons of compounds **6 b**, **6f**, **6j** and **6n** appeared as singlet signals around *δ* 2.35 *ppm*. Moreover, ^13 ^C NMR spectra of spirooxindoles **6a–p** showed signals resonating in the range *δ* 177.72–178.10 *ppm* attributable for the carbon of the carbonyl (C=O) groups, whereas the carbons of the methoxy (-OCH_3_) groups of compounds **6 g**, **6k** and **6 m–o** and carbons of the methyl (-CH_3_) groups of compounds **6 b**, **6j** and **6n** appeared as two signals around *δ* 56.0 and 21.4 *ppm*, respectively.

### Biological evaluation

#### *In vitro* anti-proliferative activity

The *in vitro* anti-proliferative activity of the newly synthesized spirooxindoles **6a–p** was examined against TNBC MDA-MB-231 cells. This assay was performed utilizing the 3–(4,5-dimethylthiazol-2-yl)-2,5-diphenyltetrazolium bromide (MTT) colorimetric assay as described by Mosmann^18^. Adriamycin was included in this assay as a reference drug. The results were expressed as median growth inhibitory concentration (IC_50_) values that represent the compounds concentrations required to afford a 50% inhibition of cell growth after 48 h of incubation, compared to untreated controls (Table 1).

**Table 1.** *In vitro* anti-proliferative activity of the newly synthesized spirooxindoles **6a–p** against MDA-MB-231 cell line.


			IC_50_ (µM)
Compound	R	Ar	MDA-MB-231
**6a**	H	C_6_H_5_-	6.70
**6b**	H	4-CH_3_-C_6_H_4_-	29.70
**6c**	H	4-OCH_3_-C_6_H_4_-	37.80
**6d**	H	4-Cl-C_6_H_4_-	12.00
**6e**	Cl	C_6_H_5_-	6.40
**6f**	Cl	4-CH_3_-C_6_H_4_-	18.20
**6g**	Cl	4-OCH_3_-C_6_H_4_-	24.10
**6h**	Cl	4-Cl-C_6_H_4_-	16.60
**6i**	Br	C_6_H_5_-	6.70
**6j**	Br	4-CH_3_-C_6_H_4_-	13.50
**6k**	Br	4-OCH_3_-C_6_H_4_-	12.70
**6l**	Br	4-Cl-C_6_H_4_-	30.60
**6m**	OCH_3_	C_6_H_5_-	17.50
**6n**	OCH_3_	4-CH_3_-C_6_H_4_-	NA^a^
**6o**	OCH_3_	4-OCH_3_-C_6_H_4_-	NA^a^
**6p**	OCH_3_	4-Cl-C_6_H_4_-	NA^a^
**Adriamycin**			0.12

a NA: Compounds having IC_50_ value >50 **µ**M.

**Figure 2.**
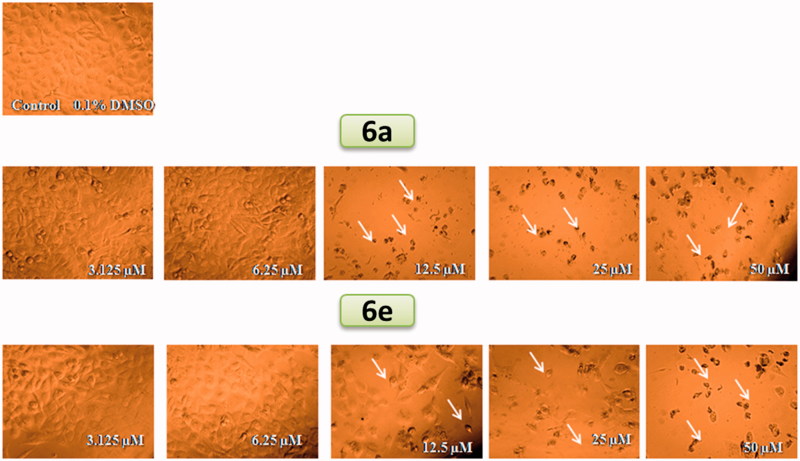
Photomicrographs showing morphological assessment of the effects of compounds **6a** and **6e** on MDA-MB-231 cell monolayers. Cells were treated with vehicle (DMSO, 0.1%) or increasing concentrations of the compounds (50, 25, 12.5, 6.25 and 3.125 µM). Arrows indicate morphological signs of cytotoxicity including cell rounding and/or disintegrated monolayer compared to the DMSO control. Photomicrographs were taken using Zeiss^®^Primovert (Carl Zeiss Microscopy GmbH, Gottingen, Germany) equipped with a digital camera. Total magnification is 150×.

As shown in Figure 2, morphological assessment using bright field phase contrast microscopy revealed adverse effects on cell morphology such as monolayer disruption in addition to cell shrinkage and rounding, as indicated by arrows.

From the obtained results, it was obvious that various prepared spirooxindoles displayed excellent to modest growth inhibitory activity against MDA-MB-231 cells. Spirooxindoles **6a**, **6e** and **6i** emerged as the most potent analogues towards TNBC MDA-MB-231 cells through this study with IC_50_ = 6.70, 6.40 and 6.70 µM, respectively. Besides, compounds **6d**, **6f**, **6 h**, **6j**, **6k** and **6 m** were moderately active against MDA-MB-231 cells with IC_50_ range (12.00 – 18.20 µM). While, spirooxindoles **6 b**, **6c**, **6 g** and **6 l** possessed weak anti-proliferative activity against MDA-MB-231 cells (IC_50_ = 29.70, 37.80, 24.10, and 30.60 µM, respectively), compounds **6n–p** did not display growth inhibitory activity against MDA-MB-231 cells.

#### Structure activity relationship (SAR)

Based on the aforementioned results of the biological anti-proliferative activity assay, important structure activity relationships could be deduced. Firstly, we investigated the impact of the C-5 substitution of the indoline moiety. The abolished activity of 5-methoxyindoline derivatives **6n–p** along with the decreased IC_50_ value of **6m**, with incorporated unsubstituted phenyl group, (17.50 µM) than that of its corresponding members **6a**, **6e** and **6i**, with unsubstituted, 5-chloro substituted and 5-bromo substituted indoline moiety, (6.70, 6.40 and 6.70 µM, respectively) indicated that unsubstitution or C-5 substitution of the indoline moiety with electron withdrawing groups (EWGs), as 5-Cl or 5-Br, is more beneficial than incorporation of electron-donating substituents, as 5-OCH_3_, to the growth inhibitory activity against MDA-MB-231 cells.

We then examined the effect of the substitution of the pendant phenyl group at C-6 of pyrazolo[3,4-*b*]pyridine moiety on the prepared spirooxindoles activities. Incorporation of unsubstituted phenyl group led to compounds **6a** (IC_50_ = 6.70 µM), **6e** (IC_50_ = 6.40 µM), **6i** (IC_50_ = 6.70 µM) and **6m** (IC_50_ = 17.50 µM) with superior activity to their corresponding 4-substituted-phenyl analogous **6c–d** (IC_50_ range: 12.00–37.80 µM), **6f–h** (IC_50_ range: 16.60–24.10 µM), **6j–l** (IC_50_ range: 12.70–30.60 µM) and **6n–p** (IC_50_ > 50 µM) against MDA-MB-231 cells, implying that incorporation of unsubstituted phenyl group is indispensable for the anti-proliferative activity.

Finally, we can deduce that the substitution pattern over the synthesized spirooxindoles is a crucial element for the anti-proliferative activity towards TNBC MDA-MB-231 cell line. Incorporation of unsubstituted or 5-EWG-substituted indoline moiety along with unsubstituted phenyl group at C-6 of pyrazolo[3,4-*b*]pyridine moiety greatly enhances the growth inhibitory activity of the target spirooxindoles **6a–p**.

#### In vitro cytotoxicity towards human normal WI-38 cells

Three potent compounds **6a**, **6e** and **6i** were evaluated for their ability to induce cytotoxic effect against human normal lung fibroblast cell line (WI-38 cells), to investigate their safety adopting the MTT assay procedures^18^. The results, expressed as (IC_50_) values, and the calculated selectivity index were displayed in Table 2.

**Table 2.** *In vitro* cytotoxic activity of compounds **6a**, **6e** and **6i** against WI-38 cells, and Selectivity index for the tested compounds.

	IC_50_ (µM)		
Compound	WI-38	MDA-MB-231	Selectivity Index
**6a**	78.1	6.7	11.7
**6e**	43.2	6.4	6.8
**6i**	39.3	6.7	5.9

The tested spirooxindoles **6a**, **6e** and **6i** showed non-significant cytotoxic effect with IC_50_ values of 78.1, 43.2 and 39.3 µM, respectively, thereby providing a high-safety profile as anti-proliferative agents with good selectivity index rang (11.7, 6.8 and 5.9 respectively).

#### Apoptosis induction in TNBC MDA-MB-231 cells

Apoptosis is a programed routine that proves to be an essential physiological process for tissue development, immune response, redundant cells clearance and homeostasis by which cells signal their own termination. Accordingly, cellular integrity is conserved by this finely tuned, self-automated death^23^. Consequently, the success of cancer cells to proliferate unconditionally is allied to its ability to halt apoptosis. Thus, targeting apoptosis induction is a successful strategy for combating tumour progression.

To further elucidate the mechanism of cell death induced by the target spirooxindoles and as a part of our ongoing efforts to develop novel pro-apoptotic agents^21^^,^^22^^,^^24–26^, we evaluated ability of compounds **6a** and **6e** to provoke apoptosis in MDA-MB-231 cells through determination of the hallmark parameters of apoptosis.

#### Effects on the levels of active caspase-3

Caspases, a family of cysteineaspartic proteases, are the crucial apoptosis mediators that provide essential links in cell regulatory networks controlling cell death. Caspase-3 is the key executioner caspase which modifies proteins ultimately responsible for apoptosis^27^. Accordingly, the effect of spirooxindoles **6a** and **6e** on the level of caspase 3 was evaluated, to give insight to the pro-apoptotic effect of the prepared spirooxindoles (Table 3).

**Table 3.** Effect of compounds **6a** and **6e** on the active caspases-3 level, and the expression levels of Bcl-2 and Bax in MDA-MB-231 cancer cells treated with each compound at its IC_50_ concentration.

Comp.	**Caspase-3** (ng/ml)	**Bax** (Pg/ml)	**Bcl-2** (ng/ml)
**6a**	0.3501 (31.5)*	405.5 (506.8)*	0.3958 (0.147)*
**6e**	0.4058 (36.5)*	353.7 (442.1)*	0.7449 (0.276)*
**Control**	0.0111	0.80	2.692

* Numbers given between parentheses are the number of folds of control.

Results in Table 3 showed that treatment of MDA-MB-231 cells with compounds **6a** and **6e** resulted in a significant elevation in the level of active caspase-3 by about 31.5 and 36.5 folds, respectively, compared to control.

#### Effects on mitochondrial apoptosis pathway (Bcl-2 family) proteins

Bcl-2 family comprises a group of crucial regulatory factors in apoptosis that finely tune the apoptotic switch on/off mechanism. Based on their functional and structural criteria, the members are divided into two major classes; group I proteins that are anti-apoptotic and group II proteins that are apoptotic. Group I anti-apoptotic proteins exert their function by inhibiting group II apoptotic proteins through simply binding to them. Group I proteins bind selectively to the active conformations of group II proteins to prevent them from being inserted into the mitochondria and thus cease the release of pro-apoptotic factors such as cytochrome c, ultimately aborting apoptosis [25]. Thus, the inhibition of group I proteins and/or the activation of group II proteins can successfully induce apoptosis. Herein, we evaluated the impact of compounds **6a** and **6e** on the level of Bcl2, as a representative group I member, and the level of Bax, as a representative group II member (Table 3).

As presented in Table 3 compound **6a** induced the protein expression of Bax with 506.8 folds of the control while 442.1 folds were recorded with compound **6e**. Parallel to this, the protein expression of the antiapoptotic marker Bcl-2 was down-regulated to 14.7% compared to that of the basal level in the control by compound **6a** while compound **6e** produced down-

#### Cell-cycle analysis

**Figure 3.**
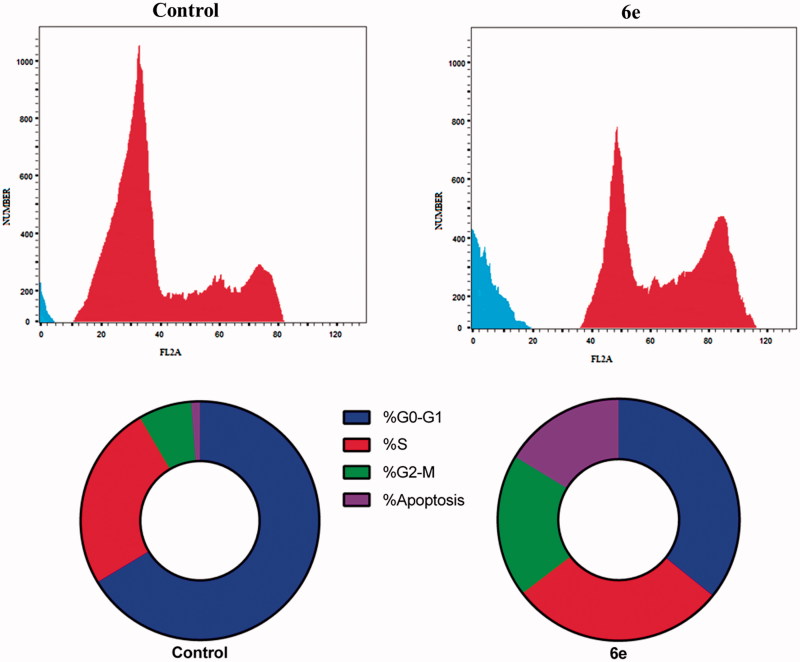
DNA-flow cytometry analysis for MDA-MB-231 cells treated with compound **6e** for 24 h at their IC_50_ concentrations. The experiments were done in triplicates.

Antiproliferative agents abort cell growth by arresting its proliferation at certain well-known checkpoints. Distinguish cells in various phases of cell cycle can be detected upon treatment of cancer cells with anticancer agents^28^. Compound **6e** was investigated for its activity to disrupt the cell cycle of MDA-MB-231 cancer cell lines. This effect was illustrated by DNA flow cytometric analysis which MDA-MB-231 cells was treated with compound **6e** at concentration equals to the IC_50_ for 24 h. Figure 3 showed that compound **6e** expressed significant decrease in the G0-G1 phase by approximately 0.5 folds related to the control. Compound **6e** displayed no significant change in the S phase while G2-M phase was arrested by 2.65 folds with 19.1% with respect to control (7.2%). Alteration of the Pre-G phase and arrest of G2-M phase were significant remarks for compound **6e** to induce apoptosis.

#### Annexin V-FITC apoptosis assay

**Figure 4.**
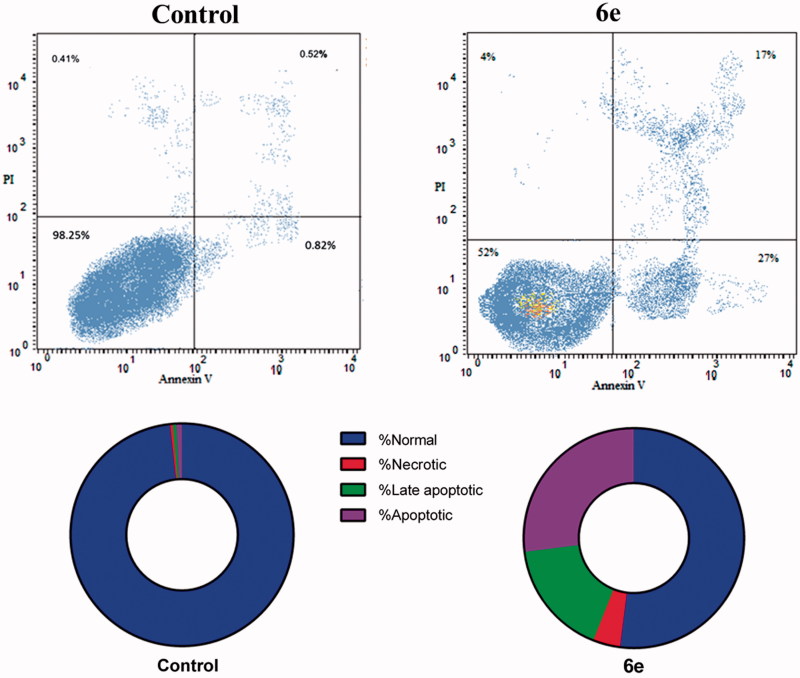
Effect of compound **6e** on the percentage of annexin V–FITC-positive staining in MDA-MB-231 cells. The four quadrants identified as: LL: viable; LR: early apoptotic; UR: late apoptotic; UL: necrotic. The experiments were done in triplicates.

Externalization of the phospholipid phosphatidylserine at the cell membrane is a one of the well-recognized hallmarks of cells going into apoptosis^17^^,^^29^. In our study, the apoptotic effect of compound **6e** was further assessed by Annexin V-FITC/PI (AV/PI) dual staining assay to examine the occurrence of phosphatidylserine externalization and also to comprehend whether cell death is due to physiological apoptosis or nonspecific necrosis (Figure 4).

Flow cytometric analysis revealed that MDA-MB-231 cells treated with compound **6e** displayed a significant increase in the percent of annexin V-FITC positive apoptotic cells (UR + LR) from 1.34% to 44% which comprises about 32.8 folds compared to control.

In conclusion, the enhanced expression of the pro-apoptotic protein Bax and the reduced expression of the anti-apoptotic protein Bcl-2 as well as the up-regulated active caspase-3 level together with a harmonized increase in the Bax/Bcl-2 ratio, highlighted that the anti-proliferative activity of the target spirooxindoles **6** might be attributed, at least in part, to the induction of the intrinsic apoptotic mitochondrial pathway.

#### In vitro EGFR kinase ELISA assay

On account of its overexpression in a significant number of TNBC, epidermal growth factor receptor (EGFR) emerged as an attractive target for developing effective therapeutic strategies for treatment of TNBC patients^28–30^. In this study the most potent anti-proliferative spirooxindoles **6a**, **6d**, **6e** and **6i–k** were selected to evaluate their potential inhibitory activity against EGFR by use of a colorimetric Enzyme-Linked Immunosorbent Assay (ELISA). Erlotinib, a clinically used EGFR inhibitor, was taken as the reference drug. The results were reported as a 50% inhibition concentration values (IC_50_) which determined as triplicate determinations from the standard curve and summarized in Table 4.

**Table 4.** IC_50_ values for the inhibitory activity of spirooxindoles **6a**, **6d**, **6e** and **6i–k** against – EGFR.

	IC_50_ (μM)^a^
Compound	EGFR
**6a**	0.43 ± 0.04
**6d**	0.36 ± 0.02
**6e**	0.15 ± 0.02
**6i**	0.12 ± 0.01
**6j**	0.51 ± 0.04
**6k**	0.31 ± 0.02
**Erlotinib**	0.11 ± 0.01

a IC_50_ values are the mean ± SD of three separate experiments.

Results revealed that the tested compounds exhibited EGFR inhibitory activity with IC_50_ values ranging from 0.12 to 0.51 μM. Compound **6i** emerged as the most potent EGFR inhibitor in this study that showed comparable potency (IC_50_ = 0.12 ± 0.01 μM) to the reference drug Erlotinib (IC_50_ = 0.11 ± 0.01 μM). Besides, compound **6e** displayed good activity (IC_50_ = 0.15 ± 0.02 μM).

## Conclusions

In summary, we have synthesized a novel series of sixteen spirooxindoles **6a–p** through a one-pot three-component reaction, with the prime aim of developing potent anti-TNBC agents. All the newly synthesized spirooxindoles **6a–p** was evaluated for their *in vitro* anti-proliferative activity towards TNBC MDA-MB-231 cells. Spirooxindoles **6a**, **6e** and **6i** were the most potent members against MDA-MB-231 cells with IC_50_ = 6.70, 6.40 and 6.70 µM, respectively. Besides, compounds **6d**, **6f**, **6h**, **6j**, **6k** and **6m** were moderately active against MDA-MB-231 cells with IC_50_ rang (12.00 – 18.20 µM). Moreover, the cytotoxicity of the active counterparts **6a**, **6e** and **6i** was examined against normal human cell line (WI-38 lung fibroblast) where none of them displayed significant cytotoxic effect, thereby providing a good safety profile. Subsequently, **6a** and **6e** were further estimated for their apoptosis induction potential. Both proved to induce apoptosis, which evidenced *via* the reduced expression of the anti-apoptotic protein Bcl-2 in addition to the enhanced expression of the pro-apoptotic protein Bax as well as the up-regulated active caspase-3 level. Moreover, **6e** displayed a significant increase in the percent of annexin V-FITC positive apoptotic cells from 1.34% to 44% which comprises about 32.8 folds compared to control. As the EGFR is frequently overexpressed in TNBC, six potent spirooxindoles was assayed for their potential inhibitory activity towards EGFR. Compounds **6e** and **6i** displayed potent inhibitory activity against EGFR with IC_50_ values of 120 and 150 nM, respectively.

## Supplementary Material

IENZ_1417276_Supplementary_Material.pdf
