# Supplementary material for: One-pot three-component synthesis of novel spirooxindoles with potential cytotoxic activity against triple-negative breast cancer MDA-MB-231 cells
Source: J Enzyme Inhib Med Chem. 2017 Dec 28;33(1):309–18. doi: 10.1080/14756366.2017.1417276 (PMC6009943; doi:10.1080/14756366.2017.1417276)

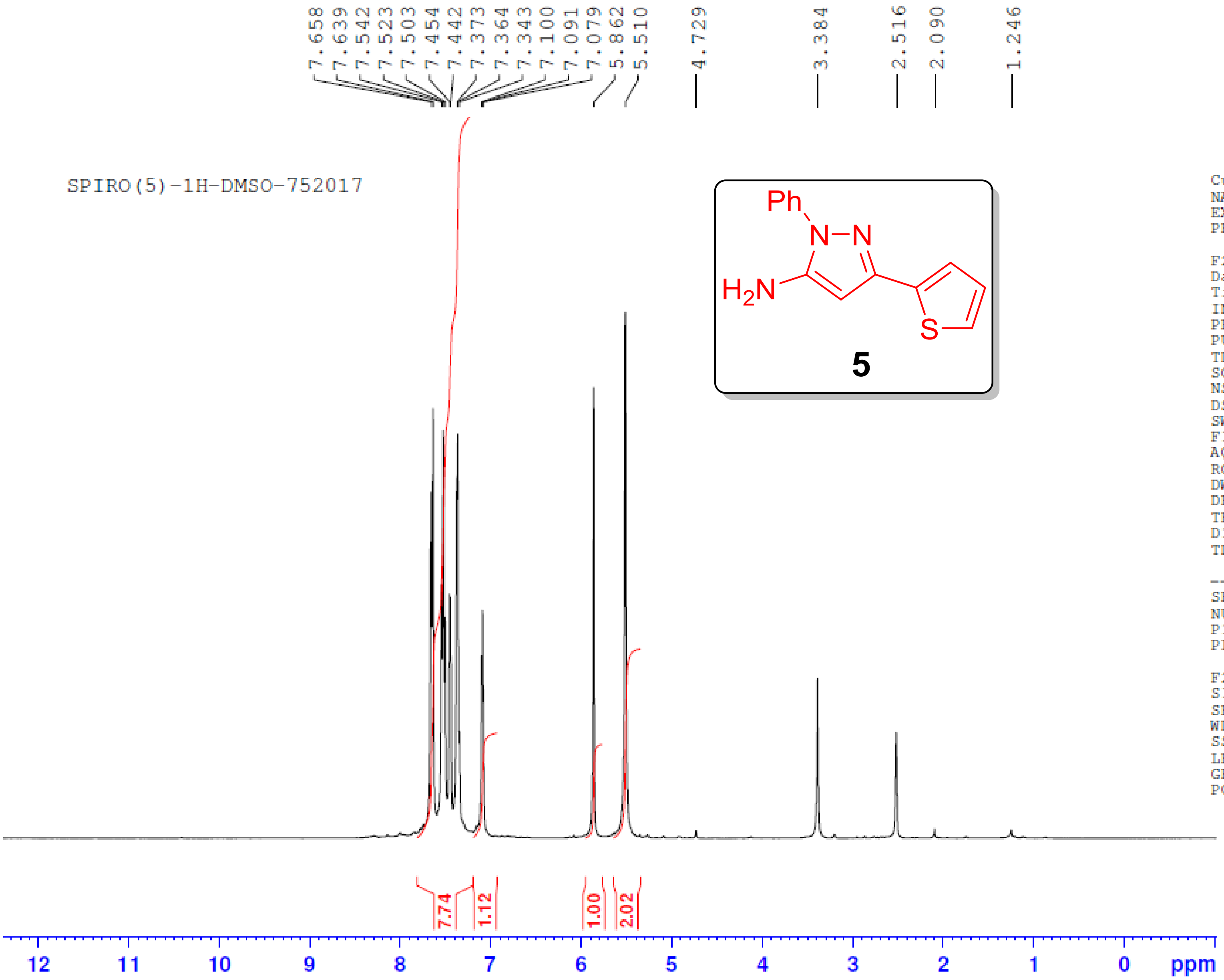

Current Data Parameters
NAME May07-2017-nmr
EXPNO 40
PROCNO 1

F2 - Acquisition Parameters
Date_ 20170507
Time 12.00
INSTRUM spect
PROBHD 5 mm PABBO BB/
PULPROG zg30
TD 65536
SOLVENT DMSO
NS 16
DS 2
SWH 8012.820 Hz
FIDRES 0.122266 Hz
AQ 4.0894465 sec
RG 30.98
DW 62.400 usec
DE 6.50 usec
TE 298.0 K
D1 1.00000000 sec
TD0 1

======== CHANNEL f1 ========
SFO1 400.1724712 MHz
NUC1 1H
P1 10.00 usec
PLW1 16.50000000 W

F2 - Processing parameters
SI 65536
SF 400.1700000 MHz
WDW EM
SSB 0
LB 0.30 Hz
GB 0
PC 1.00

SPIRO(5)-13C-DMSO-752017

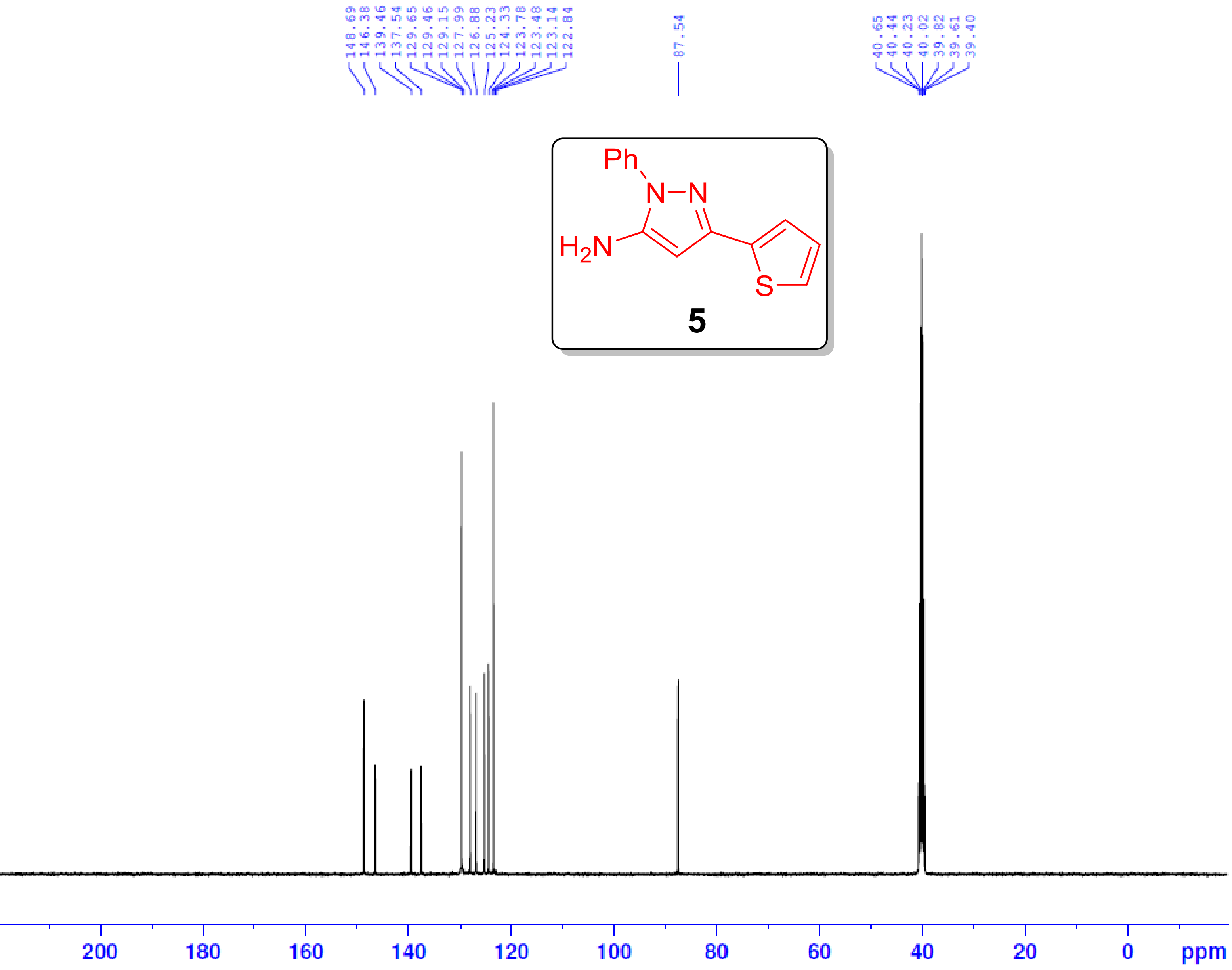

Current Data Parameters
NAME May07-2017-nmr
EXPNO 41
PROCNO 1

F2 - Acquisition Parameters
Date_ 20170507
Time 11.55
INSTRUM spect
PROBHD 5 mm PABBO BB/
PULPROG zgpg30
TD 65536
SOLVENT DMSO
NS 1024
DS 4
SWH 24038.461 Hz
FIDRES 0.366798 Hz
AQ 1.3631488 sec
RG 194.81
DW 20.800 usec
DE 6.50 usec
TE 300.0 K
D1 2.00000000 sec
D11 0.03000000 sec
TD0 1

======== CHANNEL f1 ========
SFO1 100.6328883 MHz
NUC1 13C
P1 10.00 usec
PLW1 66.00000000 W

======== CHANNEL f2 ========
SFO2 400.1716007 MHz
NUC2 1H
CPDPRG[2 waltz16
PCPD2 90.00 usec
PLW2 16.50000000 W
PLW12 0.20370001 W
PLW13 0.16500001 W

F2 - Processing parameters
SI 32768
SF 100.6228270 MHz
WDW EM
SSB 0
LB 1.00 Hz
GB 0
PC 1.40

SPIRO(5)-13C-DMSO-752017

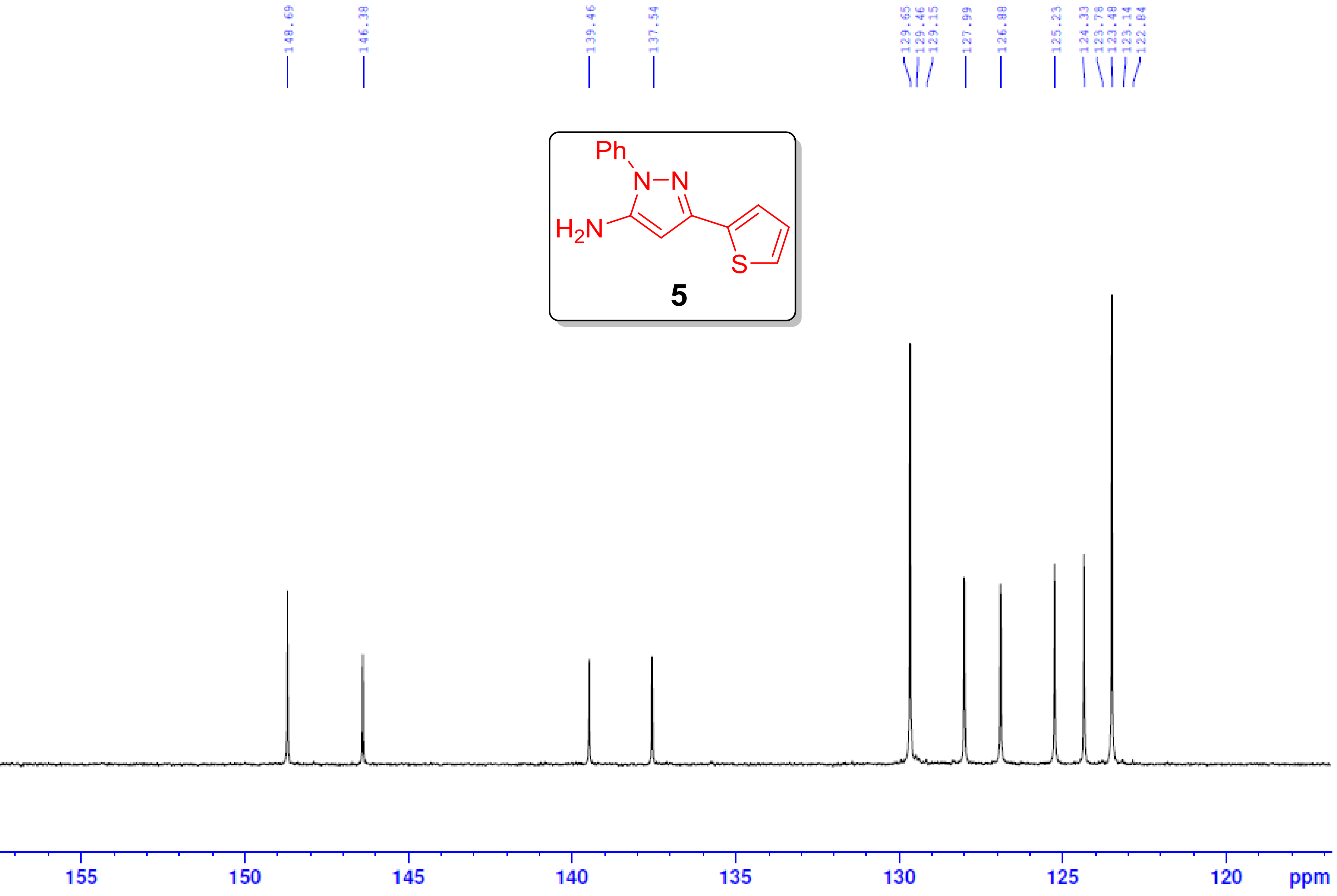

Wagdy ElDehna_H_C

Microanalytical Unit - FOPCU - NMR laboratory
www.pharma.cu.edu.eg dir-mau.fopcu@pharma.cu.edu.eg

BRUKER

```
Current Data Parameters
NAME       Wagdy ElDehna_H_C
EXPNO                    10
PROCNO                    1

F2 - Acquisition Parameters
Date_              20160126
Time                  14.19
INSTRUM               spect
PROBHD     5 mm PABBO BB/
PULPROG                zg30
TD                    65536
SOLVENT                DMSO
NS                       32
DS                        2
SWH                8012.820 Hz
FIDRES             0.122266 Hz
AQ                4.0894465 sec
RG                   129.43
DW                   62.400 usec
DE                     6.50 usec
TE                    298.1 K
D1               1.00000000 sec
TD0                       1

======== CHANNEL f1 ========
SFO1            400.1924713 MHz
NUC1                     1H
P1                    15.00 usec
PLW1            10.39999962 W

F2 - Processing parameters
SI                    65536
SF              400.1900000 MHz
WDW                      EM
SSB          0
LB                     0.30 Hz
GB           0
PC                     1.00
```

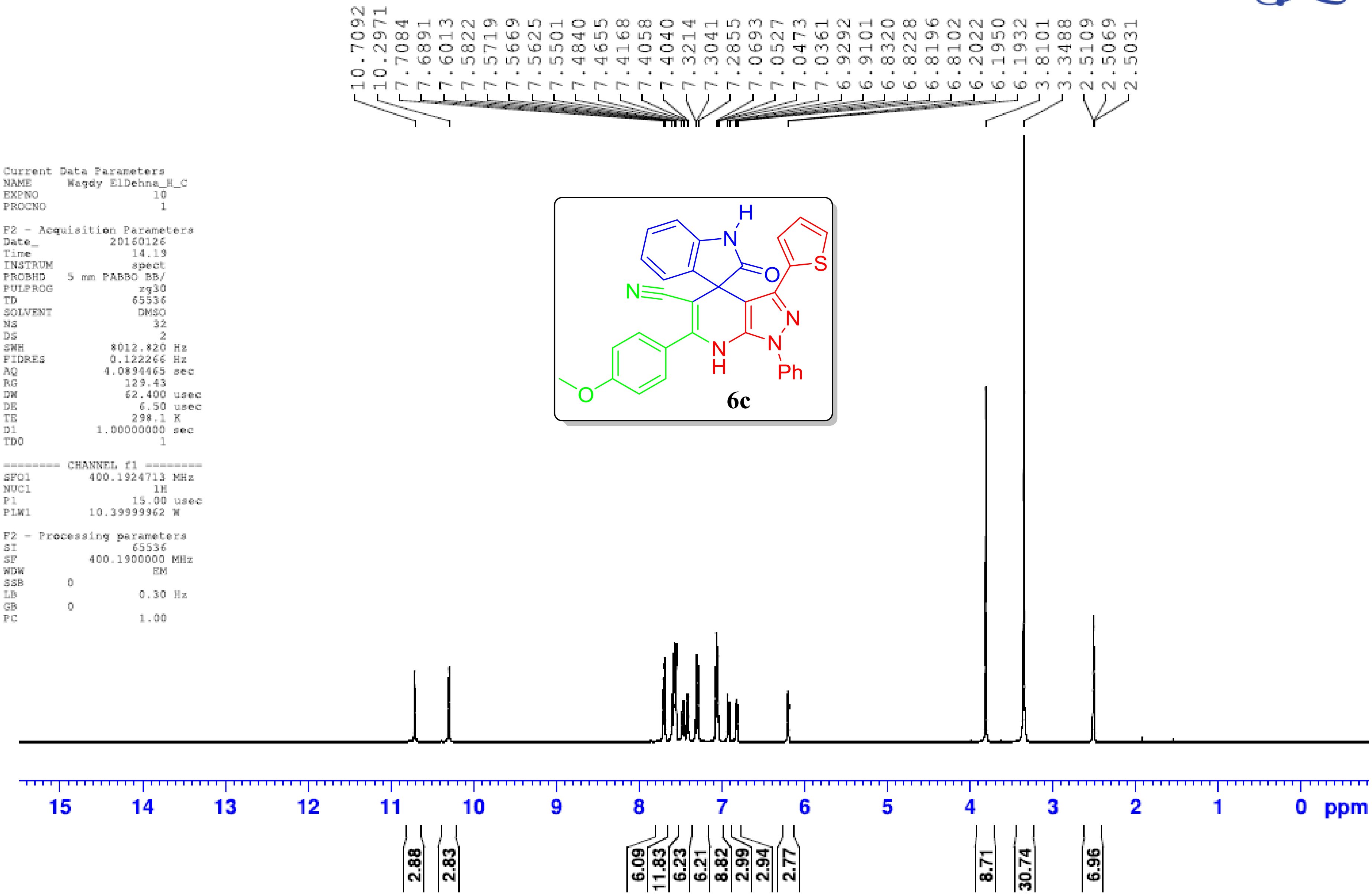

Wagdy ElDehna_H_D

Microanalytical Unit - FOPCU - NMR laboratory
www.pharma.cu.edu.eg dir-mau.fopcu@pharma.cu.edu.eg

BRUKER

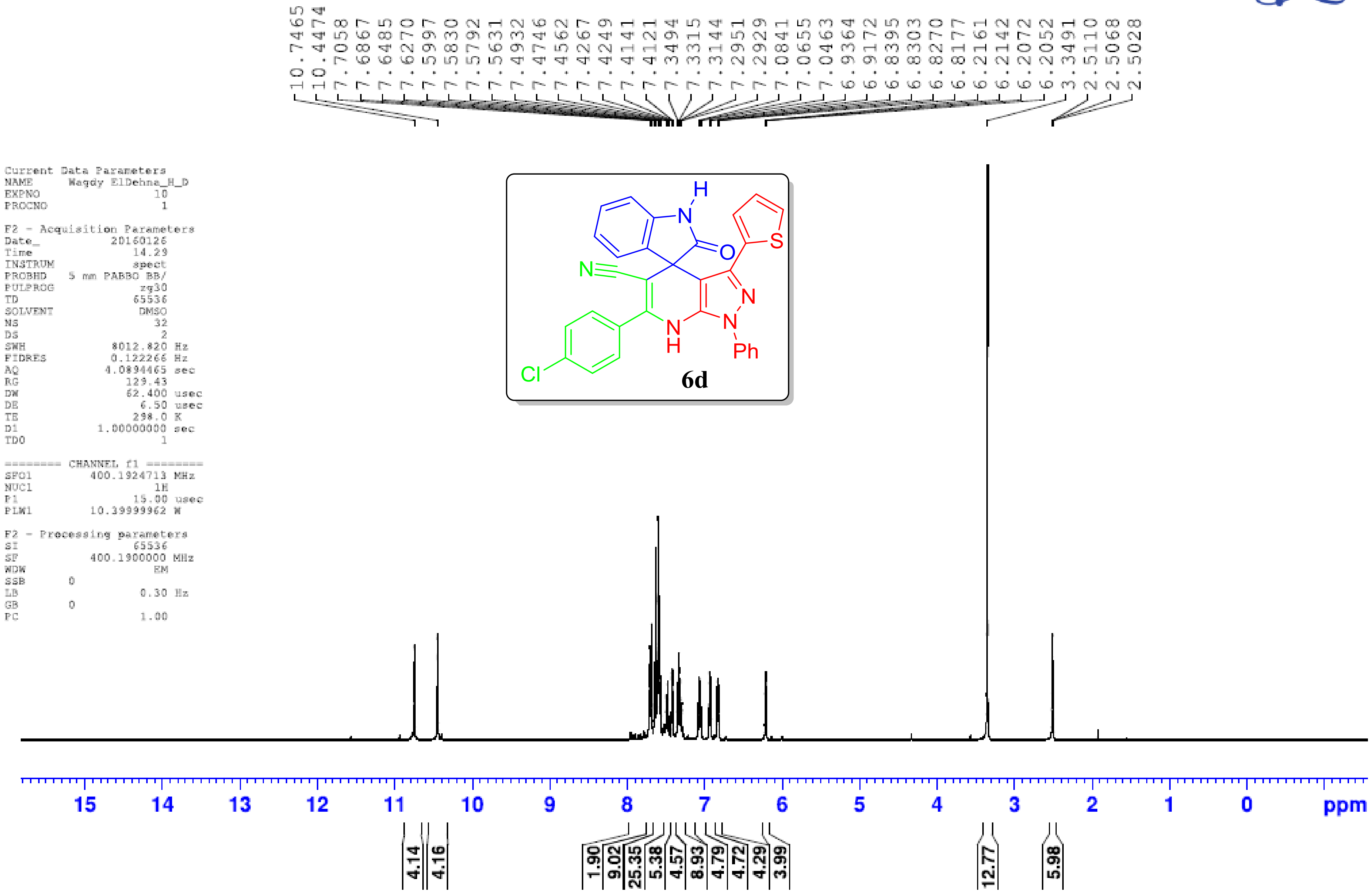

```
Current Data Parameters
NAME      Wagdy ElDehna_H_I
EXPNO                   10
PROCNO                   1

F2 - Acquisition Parameters
Date_             20160126
Time                 14.24
INSTRUM              spect
PROBHD    5 mm PABBO BB/
PULPROG               zg30
TD                   65536
SOLVENT               DMSO
NS                      32
DS                       2
SWH             8012.820 Hz
FIDRES          0.122266 Hz
AQ             4.0894465 sec
RG                  146.06
DW                  62.400 usec
DE                    6.50 usec
TE                   298.0 K
D1              1.00000000 sec
TD0                      1

======== CHANNEL f1 ========
SFO1         400.1924713 MHz
NUC1                    1H
P1                   15.00 usec
PLW1           10.39999962 W

F2 - Processing parameters
SI                   65536
SF             400.1900000 MHz
WDW                     EM
SSB      0
LB                    0.30 Hz
GB       0
PC                    1.00
```

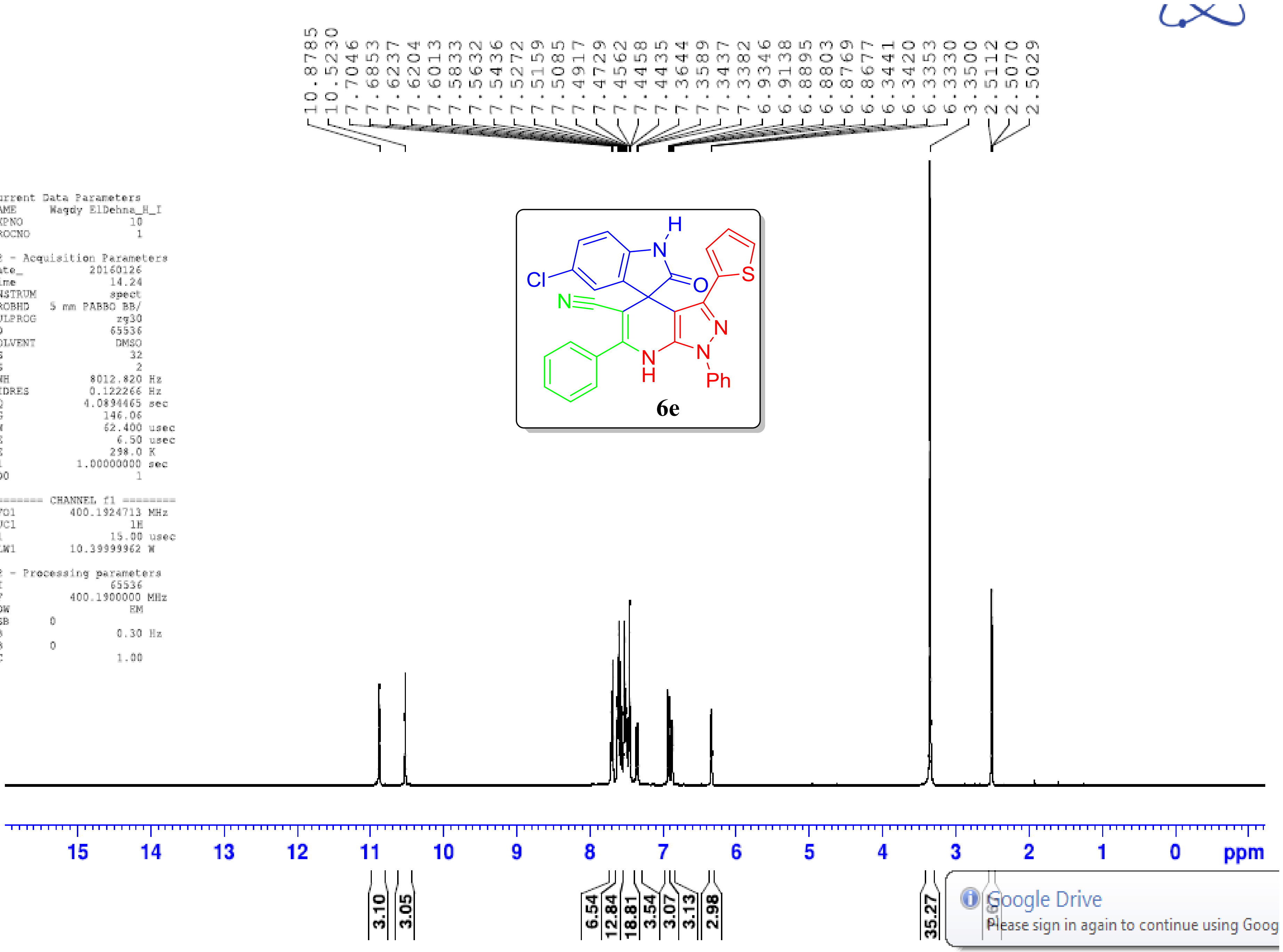

Google Drive
Please sign in again to continue using Goog

Wagdy ElDehna_H_J

Microanalytical Unit - FOPCU - NMR laboratory
www.pharma.cu.edu.eg dir-mau.fopcu@pharma.cu.edu.eg

BRUKER

```
Current Data Parameters
NAME       Wagdy ElDehna_H_J
EXPNO                     10
PROCNO                     1

F2 - Acquisition Parameters
Date_               20160126
Time                   14.14
INSTRUM                spect
PROBHD    5 mm PABBO BB/
PULPROG                 zg30
TD                     65536
SOLVENT                 DMSO
NS                        32
DS                         2
SWH               8012.820 Hz
FIDRES            0.122266 Hz
AQ               4.0894465 sec
RG                   146.06
DW                   62.400 usec
DE                     6.50 usec
TE                    298.1 K
D1              1.00000000 sec
TD0                        1

======== CHANNEL f1 ========
SFO1          400.1924713 MHz
NUC1                      1H
P1                    15.00 usec
PLW1           10.39999962 W

F2 - Processing parameters
SI                     65536
SF            400.1900000 MHz
WDW                       EM
SSB        0
LB                     0.30 Hz
GB         0
PC                      1.00
```

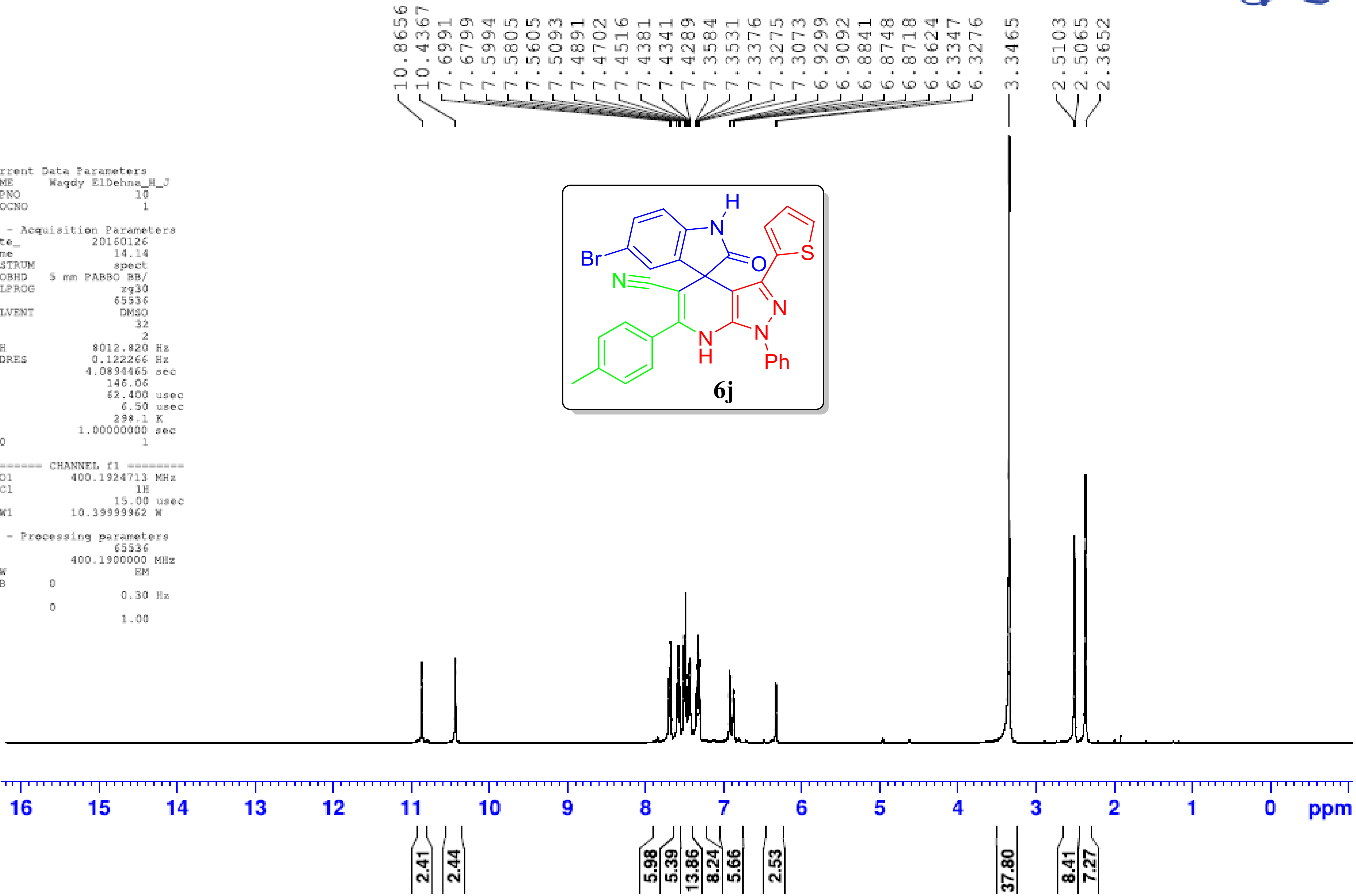

6j

Dr_WagdyMohamed-N-D2O

Sample Name	Dr_WagdyMohamed-N-D2O	Pulse sequence	PROTON	Temperature	25	Study owner	vnmr1
Date collected	2016-01-20	Solvent	dmso	Spectrometer	nmr400-mercury400	Operator	vnmr1

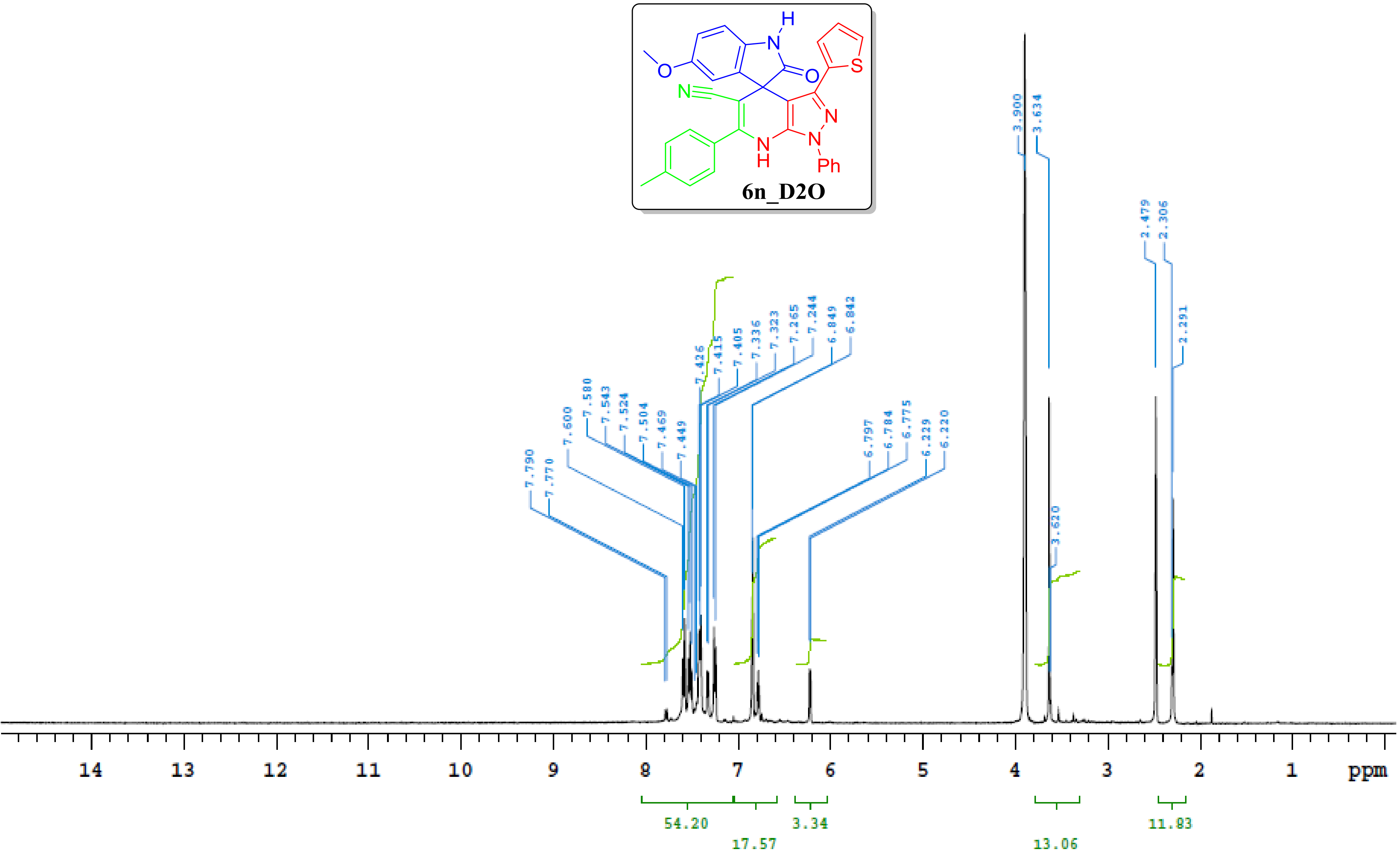

Supplement: IENZ_1417276_Supplementary_Material.pdf [file IENZ_A_1417276_SM7271.pdf]
